# Evaluation of commercial kits for isolation and bisulfite conversion of circulating cell-free tumor DNA from blood

**DOI:** 10.1186/s13148-023-01563-0

**Published:** 2023-09-14

**Authors:** Stine H. Kresse, Sara Brandt-Winge, Heidi Pharo, Bjørnar T. B. Flatin, Marine Jeanmougin, Hege Marie Vedeld, Guro E. Lind

**Affiliations:** 1 Department of Molecular Oncology, Institute for Cancer Research, Norwegian Radium Hospital, Oslo University Hospital, Montebello, 0379 Oslo, Norway; 2 Department of Biosciences, Faculty of Mathematics and Natural Sciences, University of Oslo, Oslo, Norway

**Keywords:** Liquid biopsy, Circulating cell-free tumor DNA, cfDNA, ctDNA, Plasma, cfDNA isolation, Bisulfite conversion, DNA methylation, Biomarker, ddPCR

## Abstract

**Background:**

DNA methylation biomarkers in circulating cell-free DNA (cfDNA) have great clinical potential for cancer management. Most methods for DNA methylation analysis require bisulfite conversion, causing DNA degradation and loss. This is particularly challenging for cfDNA, which is naturally fragmented and normally present in low amounts. The aim of the present study was to identify an optimal combination of cfDNA isolation and bisulfite conversion kits for downstream analysis of DNA methylation biomarkers in plasma.

**Results:**

Of the five tested bisulfite conversion kits (EpiJET Bisulfite Conversion Kit, EpiTect Plus DNA Bisulfite Kit (EpiTect), EZ DNA Methylation-Direct Kit, Imprint DNA Modification Kit (Imprint) and Premium Bisulfite Kit), the highest and lowest DNA yield and recovery were achieved using the EpiTect kit and the Imprint kit, respectively, with more than double the amount of DNA for the EpiTect kit. Of the three tested cfDNA isolation kits (Maxwell RSC ccfDNA Plasma Kit, QIAamp Circulating Nucleic Acid Kit (CNA) and QIAamp MinElute ccfDNA Mini Kit), the CNA kit yielded around twice as much cfDNA compared to the two others kits, although with more high molecular weight DNA present. When comparing various combinations of cfDNA isolation kits and bisulfite conversion kits, the CNA kit and the EpiTect kit were identified as the best-performing combination, resulting in the highest yield of bisulfite converted cfDNA from normal plasma, as measured by droplet digital PCR (ddPCR). As a proof of principle, this kit combination was used to process plasma samples from 13 colorectal cancer patients for subsequent ddPCR methylation analysis of *BCAT1* and *IKZF1*. Methylation of *BCAT1* and/or *IKZF1* was identified in 6/10 (60%) stage IV patients and 1/3 (33%) stage III patients.

**Conclusions:**

Based on a thorough evaluation of five bisulfite conversion kits and three cfDNA isolation kits, both individually and in combination, the CNA kit and the EpiTect kit were identified as the best-performing kit combination, with highest DNA yield and recovery across a range of DNA input amounts. The combination was successfully used for detection of clinically relevant DNA methylation biomarkers in plasma from cancer patients.

**Supplementary Information:**

The online version contains supplementary material available at 10.1186/s13148-023-01563-0.

## Background

Detection of biomarkers in circulating cell-free DNA (cfDNA) from liquid biopsies has great clinical potential for cancer management, including early detection, diagnosis, prognosis and monitoring of cancer recurrence. Compared to tissue biopsies, liquid biopsies are minimally invasive, cost less and provide the opportunity to monitor tumor evolution and real-time drug response as well as to reveal tumor heterogeneity [1]. cfDNA in plasma is, however, usually present in low amounts, normally 1–15 ng/ml plasma in healthy individuals and 1–150 ng/ml plasma for cancer patients [2]. Also, only a small fraction of cfDNA originates from cancer cells compared to normal cells, typically less than 2% [2]. Altogether, this makes detection of cfDNA challenging and requires use of a highly sensitive method, such as droplet digital PCR (ddPCR) that can detect down to one aberrant gene copy among 100,000 wild-type copies [3].

Aberrant DNA methylation is a promising cancer biomarker, as it is known to arise early during cancer development and tends to be highly recurrent among cancer patients [4]. Most DNA methylation analyses require bisulfite conversion of DNA, where DNA methylation patterns can be interpreted by alterations in the DNA sequence before and after bisulfite treatment. However, bisulfite conversion causes fragmentation of DNA due to harsh conditions with low pH and high temperature, resulting in loss of DNA for downstream analyses [5]. This is particularly challenging for cfDNA, which is already limited in amount and naturally fragmented. The fragment length of cfDNA from healthy individuals is typically around 166 bp, while cfDNA from cancer patients is even shorter, around 143 bp [6]. The small fragment size may result in further loss during the purification process after bisulfite conversion. Consequently, despite its promise in cancer management, detection of DNA methylation aberrations in cfDNA from blood may be challenging and places a great demand on methodology.

Methods for cfDNA isolation and bisulfite conversion prior to DNA methylation analyses have not yet been fully standardized. Several commercial kits for bisulfite conversion are available, but compared to their extensive use, relatively few comparison studies evaluating the kit performances have been performed [7–13]. For isolation of cfDNA from blood, on the other hand, the relative performance of commercial kits has been well documented [14–22]. Although more widely explored, there is currently no standard recommended method for cfDNA isolation, but the QIAamp Circulating Nucleic Acid kit is often referred to as a “gold standard” based on its consistent high ranking in terms of yield [16, 18, 22]. Identifying an optimal combination of cfDNA isolation and bisulfite conversion methods is critical to increase the success rate for downstream analyses.

The aim of this study was to identify an optimal protocol for detecting DNA methylation biomarkers in blood, by comparing the performance of five different bisulfite conversion kits and three different cfDNA isolation kits, both individually and in combination. The kits were evaluated based on DNA quantity, DNA quality, degree of DNA fragmentation, contamination of high molecular weight (HMW) DNA after cfDNA isolation and DNA recovery after bisulfite conversion. As a proof-of-principle, the best-performing kit combination was used for detection of clinically relevant DNA methylation biomarkers in blood samples from colorectal cancer patients.

## Results

**Fig. 1:**
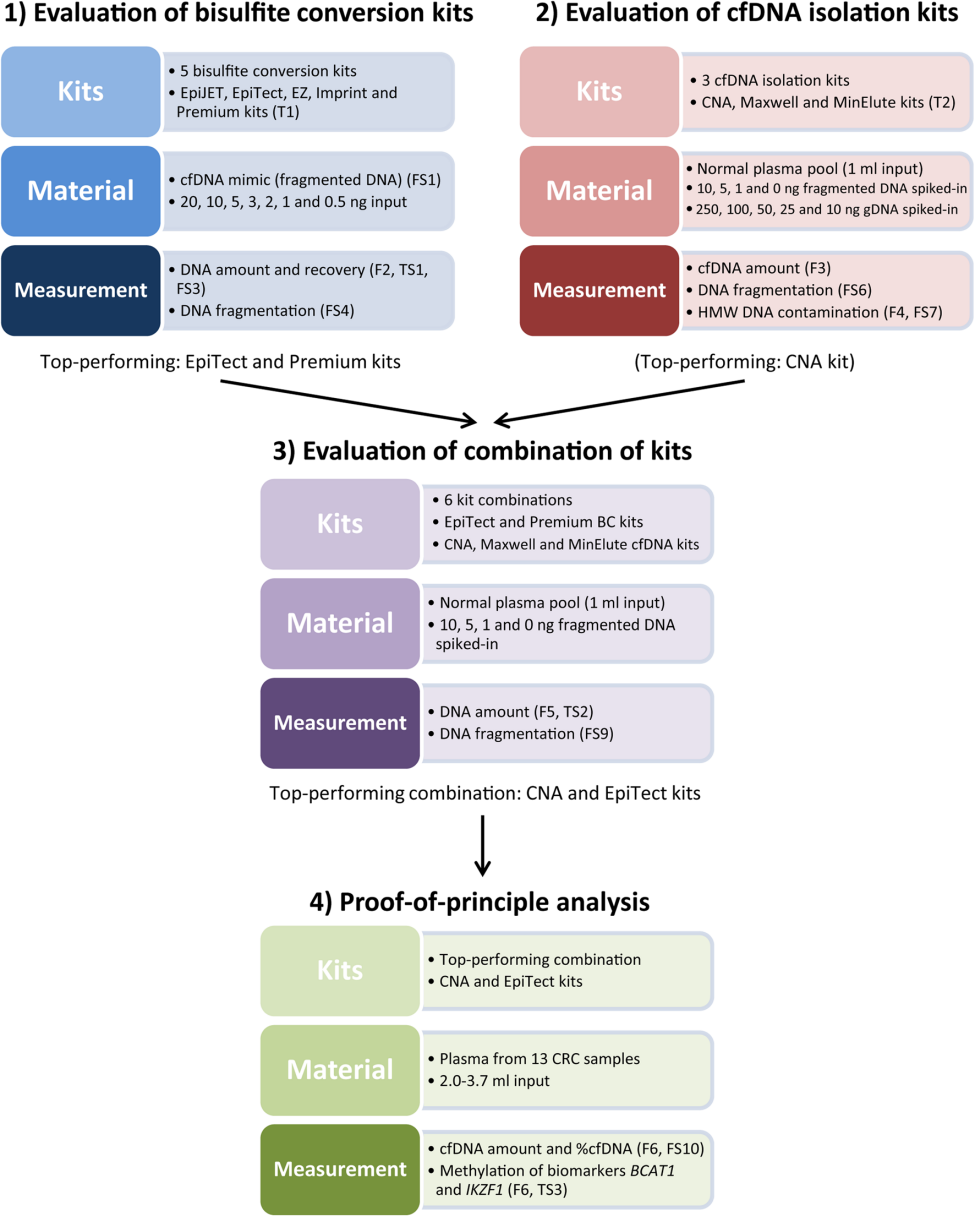
Overview of the overall workflow for this study. F: figure, T: table, FS: supplementary figure, TS: supplementary table, HMW: high molecular weight, BC: bisulfite conversion and CRC: colorectal cancer

An overview of the overall workflow of this study is shown in Fig. 1.

### Evaluation of bisulfite conversion kits

Five different bisulfite conversion kits were selected for comparison based on their consistent high performance in previous studies [7–10, 12, 13]: EpiJET Bisulfite Conversion Kit (hereafter termed EpiJET kit), EpiTect Plus DNA Bisulfite Kit (hereafter termed EpiTect kit), EZ DNA Methylation-Direct Kit (hereafter termed EZ kit), Imprint DNA Modification Kit (hereafter termed Imprint kit) and Premium Bisulfite Kit (hereafter termed Premium kit). An overview of the key characteristics of the bisulfite conversion kits is shown in Table 1, and an overview of the workflow is shown in Additional file 1: Fig. S2.

**Table 1:** Key characteristics of bisulfite conversion kits

Kit name	Manufacturer	Input amount (ng)	Optimal input amount (ng)	Input volume (µl)	Elution volume (µl)	DNA protection	Conversion temperature (°C)	Conversion time (min)	Protocol time (min)^c^	Automation
EpiJET Bisulfite Conversion	Thermo Scientific	0.05–2000	200–500	1–20	10–20	No	98 + 60	160	220	No
EpiTect Plus DNA Bisulfite	QIAGEN	1–2000	–	1–20 (40^a^)	10–15	Yes (carrier RNA)	95 + 60	300	370	Partly (clean-up QIAcube)
EZ DNA Methylation-Direct	Zymo Research	0.05–2000	200–500	1–20	10^b^	No	98 + 64	218	260	No
Imprint DNA Modification	Sigma-Aldrich	0.1–1000	50–200	1–24	8–20	Yes (BSA solution)	65	90	155	No
Premium Bisulfite	Diagenode	0.1–2000	200–500	1–20	10^b^	No	98 + 60	68	115	No

^a^Input volume can be up to 40 µl when using 1–500 ng input DNA, reducing the volume of DNA protection buffer

^b^Elution volume can be higher, no maximum volume stated

^c^Approximate time running 12 samples

**Fig. 2:**
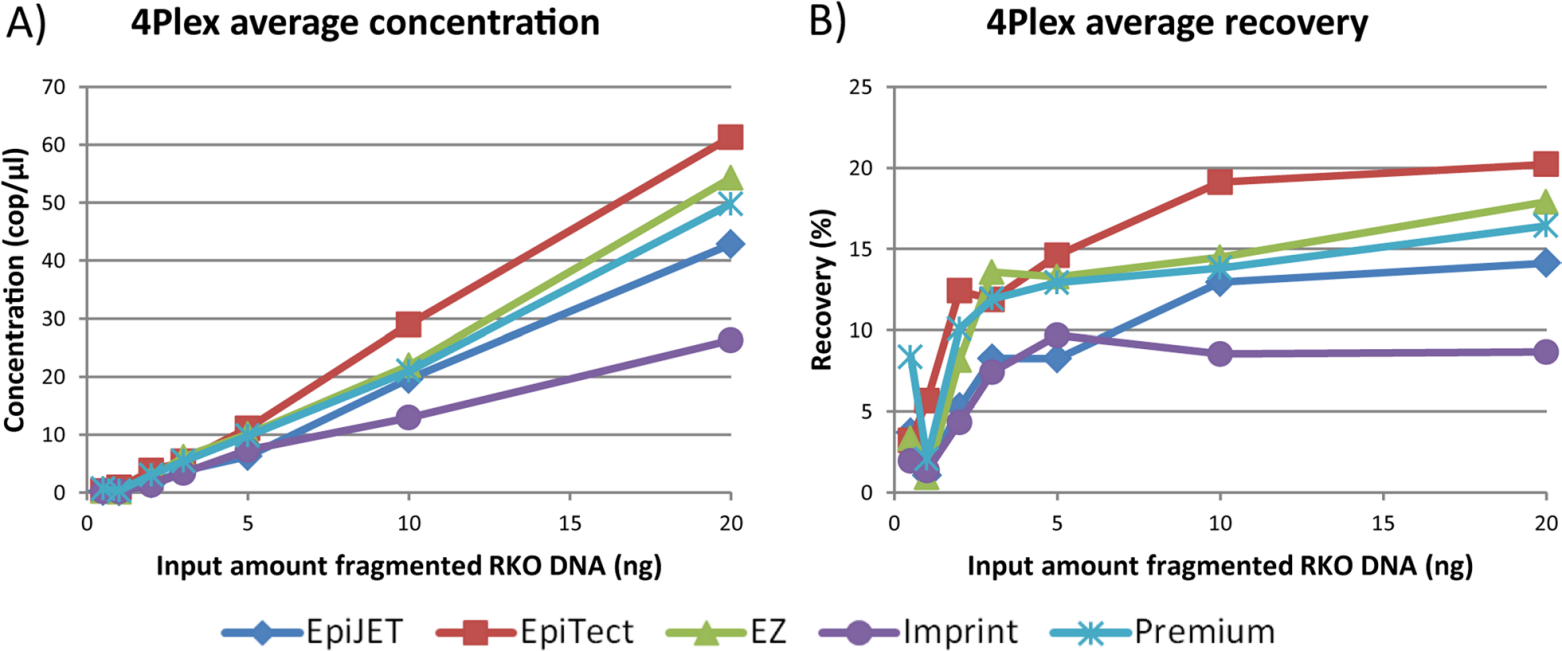
DNA quantity and DNA recovery after bisulfite conversion. **A** Average concentration of bisulfite converted DNA (cop/µl) and **B** Average recovery of DNA after bisulfite conversion (%), for all bisulfite conversion kits for the different input amounts of fragmented RKO DNA, determined by ddPCR

To quantify the total amount of bisulfite converted DNA and determine the recovery after bisulfite conversion, ddPCR was performed using the 4Plex control assay [23]. The average concentration of bisulfite converted DNA was identified for all bisulfite conversion kits for different input amounts of fragmented DNA extracted from the colon cancer cell line RKO (20–0.5 ng; Fig. 2A and Additional file 2: Table S1). The EpiTect kit resulted in the highest DNA concentrations in general across all input amounts, closely followed by the Premium and the EZ kits. Comparing the Premium and EZ kits specifically, the former performed slightly better in the lower range of DNA input (2–0.5 ng), and the latter performed slightly better in the higher range of DNA input (20–3 ng). The Imprint kit and EpiJET kit resulted in the lowest concentrations for all input amounts. The average DNA recovery after bisulfite conversion was further determined (Fig. 2B and Additional file 2: Table S1). For the EpiTect and Premium kits, the recovery was between 10 and 20% for input amounts down to 2 ng, but dropped to less than 10% with an input of ≤ 1 ng DNA. For the Imprint kit, the recovery was below 10% also for the higher input amounts. The total amount of bisulfite converted DNA was also quantified using the *MYOD1* control assay [10], and the results were in line with those observed using the 4Plex control (Additional file 1: Fig. S3 and Additional file 2: Table S2).

The bisulfite conversion kits were ranked and scored according to highest average concentration of bisulfite converted DNA for all tested input amounts (Additional file 2: Table S1). The summarized scores demonstrated that the EpiTect and Premium kits performed better overall than the other kits in terms of concentration of bisulfite converted DNA and recovery of DNA after bisulfite conversion.

The fragment length distribution of the bisulfite converted DNA was assessed for all bisulfite conversion kits by electrophoretic analysis. For input amounts of 20 and 10 ng DNA, the EpiTect kit showed the highest average peak fragment lengths, followed by the EZ, EpiJET and Premium kits (Additional file 1: Fig. S4). A similar pattern was also observed for lower amounts of input DNA (data not shown).

Based on an overall assessment of DNA quantity and recovery across a range of input amounts, as well as the degree of DNA fragmentation, the EpiTect and Premium kits were identified as the best-performing bisulfite conversion kits and were selected for further evaluation in combination with the cfDNA isolation kits.

### Evaluation of cfDNA isolation kits

Three different cfDNA isolation kits were selected for comparison based on their consistent high performance in previous studies [14–17]; QIAamp Circulating Nucleic Acid Kit (hereafter termed CNA kit), QIAamp MinElute ccfDNA Mini Kit (hereafter termed MinElute kit) and Maxwell RSC ccfDNA Plasma Kit (hereafter termed Maxwell kit). An overview of the key characteristics of the cfDNA isolation kits is shown in Table 2, and an overview of the workflow is shown in Additional file 1: Fig. S5.

**Table 2:** Key characteristics of cfDNA isolation kits

Kit name	Manufacturer	Input volume (ml)	Elution volume (µl)	Clean-up type	Protocol time (min)^b^	Automation
Maxwell RSC ccfDNA Plasma	Promega	0.2–1	60	Magnetic beads	70	Yes (Maxwell RSC)
QIAamp Circulating Nucleic Acid	QIAGEN	1–5	20–150	Spin column	90	Partly (clean-up QIAcube)
QIAamp MinElute ccfDNA Mini	QIAGEN	1–4^a^	20–80	Magnetic beads + spin column	70	Partly (clean-up QIAcube)

^a^Input volume 4–10 ml using the QIAamp MinElute ccfDNA Midi Kit

^b^Approximate time running 12 samples

**Fig. 3:**
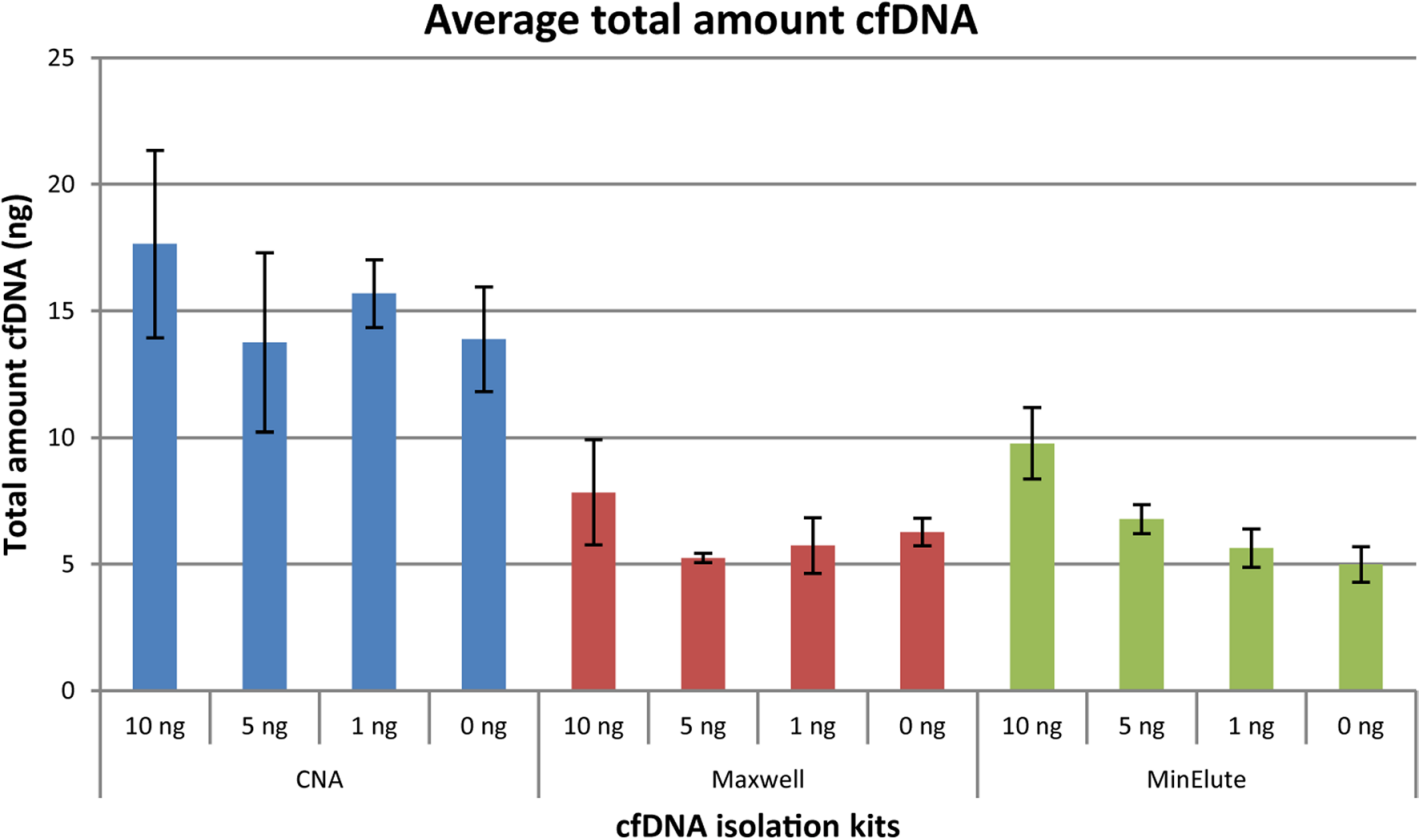
cfDNA quantity after cfDNA isolation. Average total amount of isolated cfDNA (ng) for all cfDNA isolation kits for the different amounts of spiked-in fragmented RKO DNA to the normal plasma pool, determined by fluorometric quantification

The average total amount of isolated cfDNA was determined for all cfDNA isolation kits for different amounts of fragmented RKO DNA (10–1 ng) spiked-in to 1 ml of the normal plasma pool, as well as plasma only (Fig. 3). The CNA kit showed the highest total amount of isolated cfDNA by fluorometric quantification, ranging from 13.9 ng for the plasma only sample to 17.6 ng for the plasma added 10 ng fragmented RKO sample. The Maxwell kit and MinElute kit showed lower yields for all amounts of spiked-in fragmented RKO DNA, ranging from 5.2–7.8 ng for the Maxwell kit and 5.0–9.8 ng for the MinElute kit. The DNA concentrations determined by the Bioanalyzer system were in concordance with the Qubit measurements, showing a higher yield of isolated cfDNA for the CNA kit than the other two kits (data not shown).

The fragment length distribution of the isolated cfDNA was assessed for all cfDNA isolation kits by electrophoretic analysis. The Maxwell and MinElute kits showed both an average peak size between 174 and 177 bp, whereas the CNA kit showed a slightly lower average peak size between 165 and 170 bp (Additional file 1: Fig. S6).

**Fig. 4:**
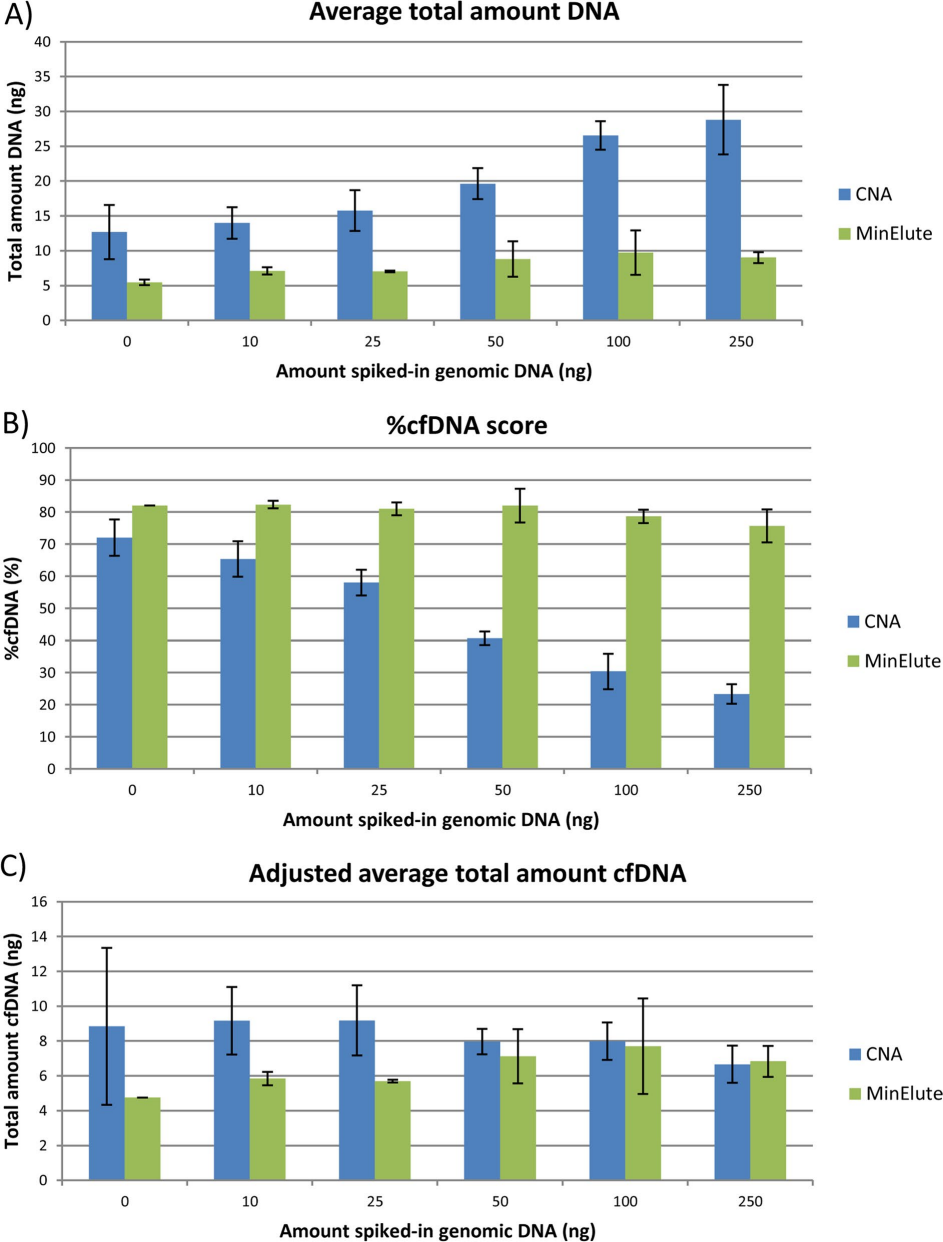
Contamination of HMW DNA after cfDNA isolation. **A** Average total amount of isolated DNA (ng) determined by fluorometric quantification, **B** Average %cfDNA score (%) determined by electrophoretic analysis and **C** Average total amount of isolated cfDNA with fragment size 50–700 bp (ng), for the CNA and MinElute kits for the different amounts of spiked-in genomic DNA to the normal plasma pool

The degree of contamination of HMW DNA in the cfDNA isolation was further examined for the CNA and MinElute kits, as the CNA kit almost doubled the yields compared to both MinElute and Maxwell kits. For isolation of cfDNA, 1 ml of the normal plasma pool was used with a range of spiked-in human non-fragmented genomic DNA (250–10 ng), as well as plasma only. The average total amount of isolated DNA was determined by fluorometric quantification for the CNA and MinElute kits for the different amounts of spiked-in genomic DNA to the normal plasma pool (Fig. 4A). Both kits showed a slight increase in total amount of isolated DNA for the addition of up to 25 ng genomic DNA. When adding ≥ 50 ng genomic DNA, a steep increase in total amount of isolated DNA was observed for the CNA kit, indicating more contamination with HMW DNA. Still, when 250 ng genomic DNA was added to the normal plasma pool, only about 10% of the genomic DNA was extracted with the CNA kit. For the MinElute kit, less than 4% of the added 250 ng genomic DNA was retained after isolation.

The %cfDNA score was further assessed by electrophoretic analysis using the Cell-free DNA ScreenTape, and the average %cfDNA was determined (Fig. 4B and Additional file 1: Fig. S7). The normal plasma pool itself showed a %cfDNA score of 72 and 82% for the CNA and MinElute kits, respectively. When adding genomic DNA to the normal plasma pool, the %cfDNA dropped markedly more for the CNA kit, down to 23% when adding 250 ng genomic DNA. However, when combining the concentration and %cfDNA measurements to determine the total amount of cfDNA with fragment size 50–700 bp, more cfDNA was in general isolated with the CNA kit compared to the MinElute kit (Fig. 4C).

Based on DNA quantity, degree of DNA fragmentation and contamination of HMW DNA after cfDNA isolation, the CNA kit was identified as the best-performing cfDNA isolation kit. However, all three cfDNA isolation kits were further evaluated in combination with the two top-performing bisulfite conversion kits in order to identify the optimal combination of kits.

### Evaluation of combination of cfDNA isolation kits and top-performing bisulfite conversion kits

The isolated cfDNA from 1 ml of the normal plasma pool with different amounts of spiked-in fragmented RKO DNA (10–0 ng) from the three cfDNA isolation kits were bisulfite converted using the two top-performing bisulfite conversion kits EpiTect and Premium, giving six different combinations of cfDNA isolation and bisulfite conversion kits. An overview of the workflow is shown in Additional file 1: Fig. S8.

**Fig. 5:**
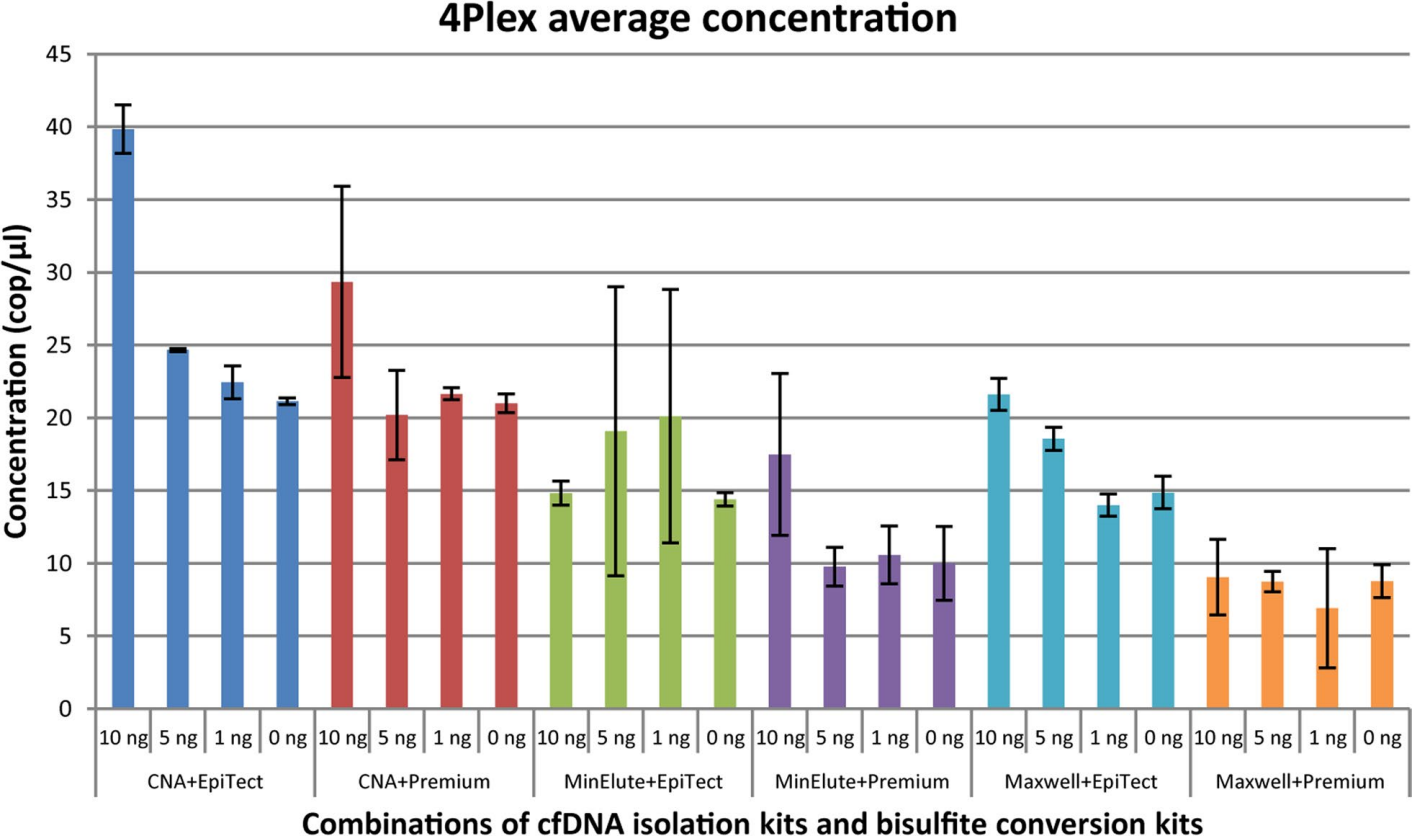
cfDNA quantity after bisulfite conversion. Average concentration of bisulfite converted cfDNA (cop/µl) for all combinations of cfDNA isolation and bisulfite conversion kits for the different amounts of spiked-in fragmented RKO DNA to the normal plasma pool, determined by ddPCR

To quantify the total amount of bisulfite converted cfDNA after cfDNA isolation and bisulfite conversion, ddPCR was performed using the 4Plex control assay [23]. The average concentration of bisulfite converted cfDNA was identified for all combinations of cfDNA isolation and bisulfite conversion kits, for the different amounts of spiked-in fragmented RKO DNA to the normal plasma pool (Fig. 5). The CNA kit in combination with both EpiTect and Premium kits showed the highest DNA concentrations, with slightly higher levels for the EpiTect kit, whereas all combinations with the Maxwell and MinElute kits gave lower concentrations. The kit combinations were ranked and scored according to highest average concentration of bisulfite converted cfDNA for all amounts of spiked-in fragmented RKO DNA to the normal plasma pool (Additional file 2: Table S3). The summarized scores showed that the CNA and EpiTect kit combination performed better than the other kit combinations in terms of amount of cfDNA after cfDNA isolation and bisulfite conversion.

The fragment length distribution of the bisulfite converted cfDNA was assessed for all combinations of cfDNA isolation and bisulfite conversion kits by electrophoretic analysis (Additional file 1: Fig. S9). The EpiTect kit showed slightly higher peak fragment lengths than the Premium kit for all combinations with cfDNA isolation kits, in concordance with the previous results (Additional file 1: Fig. S4). Although there was a slight difference in the average peak fragment length of isolated cfDNA for the three cfDNA isolation kits (Additional file 1: Fig. S6), there were no differences in average peak fragment lengths between the cfDNA isolation kits after bisulfite conversion.

Based on these results, the CNA kit and EpiTect kit were identified as the best-performing kit combination among the six different cfDNA isolation and bisulfite conversion kit combinations tested.

### Detection of DNA methylation biomarkers in blood samples from colorectal cancer patients using top-performing kit combination

**Fig. 6:**
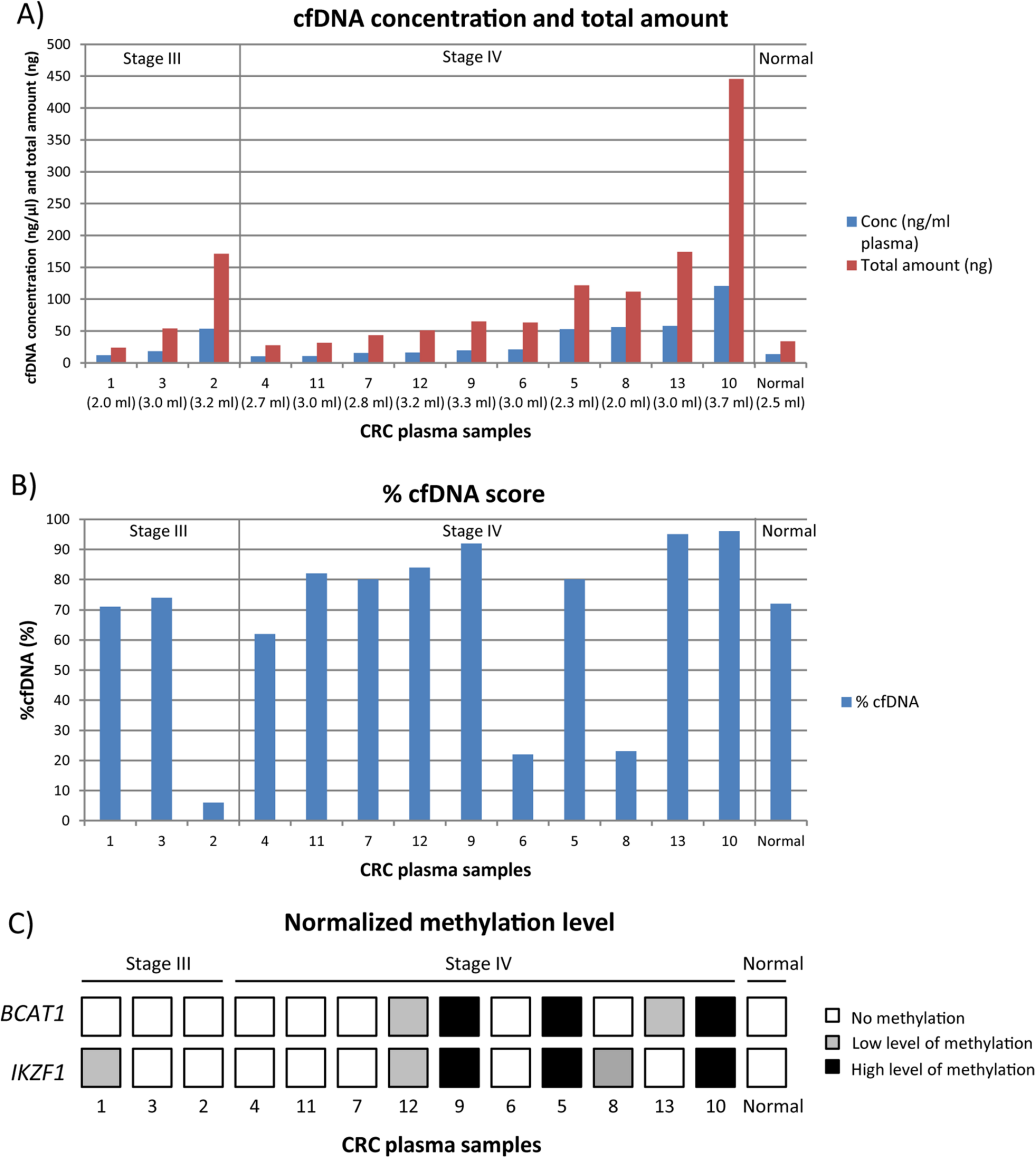
cfDNA quantity and biomarker DNA methylation levels. **A** Concentration (ng/ml plasma) and total amount (ng) determined by fluorometric quantification and **B** %cfDNA score (%) determined by electrophoretic analysis for isolated cfDNA from plasma for 13 colorectal cancer patients. **C** Normalized DNA methylation levels of *BCAT1* and *IKZF1* for 13 colorectal cancer patients, determined by ddPCR. CRC; colorectal cancer

The top-performing combination of the CNA and EpiTect kits was used for isolation and bisulfite conversion of cfDNA from plasma from 13 colorectal cancer patients with stage III (*n* = 3) and stage IV (*n* = 10) tumors. cfDNA was successfully obtained for all samples with concentrations varying between 10 and 58 ng/ml plasma for the majority of the samples by fluorometric quantification (Fig. 6A). One sample gave as much as 120 ng/ml plasma. There was no difference in cfDNA yield regarding the tumor stage, and no concordance between the total amount of cfDNA obtained and the amount of input plasma. The fragment length distribution and %cfDNA score of the isolated cfDNA was further assessed by electrophoretic analysis, showing large variations in %cfDNA between the samples (Fig. 6B and Additional file 1: Fig. S10). The %cfDNA score varied between 6 and 96%, but the vast majority of samples had at least 70% cfDNA. There were no overall concordance between the %cfDNA score and the amount of cfDNA isolated.

The isolated cfDNA from the 13 colorectal cancer patients were further bisulfite converted using the EpiTect kit, with all available amount of cfDNA as input. The DNA methylation level of two biomarkers used for the detection of recurrence of colorectal cancer, *BCAT1* and *IKZF1* [24, 25], was analyzed using ddPCR with the 4Plex assay for normalization (Fig. 6C). DNA methylation of *BCAT1* and/or *IKZF1* was identified in 7/13 samples, of which six were stage IV samples. Thus, 6/10 (60%) stage IV samples showed DNA methylation of one or both biomarkers. Three of the stage IV samples showed high DNA methylation levels of both *BCAT1* and *IKZF1*, whereas the other three stage IV samples and one stage III sample showed low levels of DNA methylation of mainly one biomarker. For the normal plasma pool, no DNA methylation was detected. A comparison of the amount of isolated cfDNA, the %cfDNA score and the detection of DNA methylation of *BCAT1* and/or *IKZF1* revealed that colorectal cancer samples positive for *BCAT1* and/or *IKZF1* DNA methylation had a %cfDNA score of at least 23% and an input amount of at least 12 ng isolated cfDNA (8.5 ng cfDNA with fragment size 50–700 bp, adjusted using the %cfDNA score) for bisulfite conversion and ddPCR (Additional file 2: Table S4). Thus, the results demonstrate the successful use of the top-performing combination of CNA and EpiTect kits for detection of these biomarkers in blood from colorectal cancer patients.

### Discussion

Detection of DNA methylation biomarkers in cfDNA from blood and other body fluids has great clinical potential for cancer management, but only a minority of blood-based liquid biopsy tests has so far been implemented in the clinic. One of the main obstacles is the scarce amount of cfDNA present in blood, which places a great demand on methodology. In the present study, we performed an extensive comparison of several commercial cfDNA isolation and bisulfite conversion kits, and identified the most optimal kit combination in terms of cfDNA yield and quality for subsequent ddPCR analyses of DNA methylation biomarkers. We demonstrate that the CNA kit gave more than double the amount of cfDNA compared to the two other kits tested, and further that more than twice as much DNA was recovered by the EpiTect kit after bisulfite conversion compared to the Imprint kit. These differences may impact the downstream analyses, underscoring the value of careful selection of kits. The combination of the CNA and EpiTect kits was shown to give highest yields of bisulfite converted cfDNA from normal plasma by ddPCR, and as a proof-of-principle, this kit combination was successfully used for detecting two DNA methylation biomarkers in a small series of colorectal cancer patients.

Bisulfite conversion is known to cause significant degradation of DNA, and the DNA recovery rate can be as low as 9–27% even for high input amounts, depending on the kits used [8, 10, 13, 26]. For scarce DNA sources such as cfDNA in plasma, limiting the DNA loss during processing is particularly crucial. We confirm that even for the best-performing bisulfite conversion kits, recovery rates are as low as 10–20% for input amounts in the higher range (up to 20 ng). For the lower range of input amounts, also touching the kit limits, the recovery rate dropped markedly and the variability among technical replicates increased. Our results are in accordance with previous studies [11, 12, 26].

Since bisulfite conversion of DNA is a chemical reaction under harsh conditions, there is a balance between the desired outcome (conversion of cytosines into uracils) and the undesired outcome (degradation of DNA and inappropriate conversion of methylated cytosines to uracils). The conversion efficiency of the bisulfite reactions has not been investigated in this study, since previous studies have shown in general a high conversion rate for the bisulfite kits utilized here, between 98 and 100% conversion [7, 9, 10, 12, 13]. Recently, enzymatic conversion of DNA has been suggested as an alternative to bisulfite conversion. Results so far show that enzymatic conversion might have advantages in terms of minimizing the damage to DNA, particularly important for high-throughput sequencing [27–29], but may also result in lower conversion efficiency and recovery rates [13]. Thus, whether enzymatic conversion is more or less advantageous compared to bisulfite conversion seem to depend on the type of downstream analysis, and remains to be more thoroughly explored.

In the present study, we have used ddPCR and the 4Plex control assay to measure DNA concentrations after bisulfite conversion. Unlike fluorometric-based concentration measurements, which only measure the presence of DNA fragments, ddPCR takes into account the amplificability of the DNA fragments and will thus be more accurate since only the DNA fragments available for amplification is quantified. Before bisulfite conversion, however, the fragmented RKO DNA was measured by fluorometric quantification, which may impact the recovery values. Nevertheless, we expect all kits to be affected in a similar manner, therefore not impacting the ranking. In addition, we have previously demonstrated that the 4Plex assay show good concordance with fluorometric quantification by Qubit assays (unpublished data). The use of the 4Plex assay for normalization has been shown to reduce the variability in methylation values, correct for differences in template amount and diminish the effect of chromosomal aberrations [23]. Results obtained using an alternative internal control to the 4Plex, *MYOD1* [10], were comparable.

For isolation of cfDNA from plasma, important parameters are the yield of cfDNA, the size of the isolated fragments and the quality in terms of amplificability of the fragments. The fragment size distribution was comparable for all three kits evaluated, but a more pronounced difference was observed in terms of cfDNA yield. The CNA kit gave approximately double the amount of cfDNA compared to the other two kits, which is in accordance with previous reports showing consistent high ranking of the CNA kit in terms of yield [16, 18, 22]. However, the higher yields of cfDNA obtained with the CNA kit gave rise to the question of whether the CNA kit also extracts more HMW DNA. The CNA kit uses a column-based clean-up in the cfDNA isolation, whereas the Maxwell and MinElute kits include a size-selection with magnetic beads. Indeed, addition of genomic DNA in various amounts to the normal plasma pool before cfDNA isolation revealed that more HMW DNA was retained using the CNA kit compared to the MinElute kit. However, when adjusting the measured DNA yield to the fraction of cfDNA in the sample (%cfDNA score), the CNA kit still resulted in higher yields of cfDNA compared to the MinElute kit, especially in the presence of lower amounts of HMW DNA. Whether the presence of HMW DNA in the background of cfDNA causes problems depends on the downstream analyses. For ddPCR reactions, which discriminate particularly well between target and background, the highest possible amount of cfDNA is preferable, even though more HMW DNA is present. For other applications, the presence of HMW DNA may be more challenging.

The CNA kit was further used for isolation of cfDNA from plasma from 13 colorectal cancer patients, and cfDNA was successfully obtained from all samples. A highly variable level of HMW DNA contamination was observed among samples, with %cfDNA scores ranging from 6 to 96%. This indicates that contamination of HMW DNA reflects the plasma sample quality before isolation. A natural source of HMW DNA could be hemolysis, often visible by a plasma color change. However, none of the plasma samples in the present study revealed signs of this. Previous studies have demonstrated the impact of using two centrifugation steps during blood processing in order to reduce HMW DNA contamination [30–32]. The single centrifugation step used for plasma separation in the present study might thus represent a limitation, as the %cfDNA scores may have been improved by incorporating a two-step centrifugation protocol. Still, at least 70% cfDNA was obtained for the vast majority of plasma samples. Of note, there was no clear correlation between positive detection of the biomarkers and the purity of the isolated cfDNA indicated by the %cfDNA score.

The biomarkers used for the proof-of-principle analysis, DNA methylation of *BCAT1* and *IKZF1*, have previously been used to detect recurrence of colorectal cancer in plasma samples [24, 25]. These biomarkers have been shown to have low background levels in blood, and a sensitivity of 63% and specificity of 98% when detecting recurrences in stage IV colorectal cancer [24]. A similar sensitivity was observed in this study, with methylation of *BCAT1* and/or *IKZF1* detected in plasma from 6/10 (60%) stage IV patients, demonstrating the successful use of the combination of CNA and EpiTect kits for detection of these biomarkers in blood from colorectal cancer patients.

### Conclusions

Based on a thorough evaluation of five bisulfite conversion kits and three cfDNA isolation kits, the CNA and the EpiTect kits were identified as the best-performing kit combination, yielding the highest average DNA concentration and recovery across a range of DNA input amounts. This combination was successfully used for detection of clinically relevant DNA methylation biomarkers in plasma from colorectal cancer patients, confirming that these kits are well suited for such analyses.

## Materials and methods

### Samples

DNA from the colon cancer cell line RKO (CRL-2577, American Type Culture Collection; ATCC) was used as input DNA for the bisulfite conversion kits. In order to mimic the size distribution of cfDNA, the RKO DNA was fragmented by sonication, generating a distribution of fragments where 95% were between 50 and 700 bp (%cfDNA score 95) and with a peak size of 165 bp. The size distribution was assessed using the Agilent 2200 TapeStation system with the D1000 ScreenTape and the Cell-free DNA ScreenTape Assays (Agilent) (Additional file 1: Fig. S1). A large pool of fragmented RKO DNA was made, sufficient for all downstream analyses.

Normal plasma was obtained from blood samples from eight voluntary healthy donors. Blood was collected using BD Vacutainer K2E (EDTA) Plus Blood Collection Tubes (Becton Dickinson) and processed within two hours. Plasma was separated from the cellular fraction by centrifugation at 1600 g for 10 min at 4 °C. The plasma samples from all donors were mixed to generate a normal plasma pool, which was further divided in 1 ml aliquots and stored at − 70 °C. Fragmented RKO DNA and Human Genomic DNA (Promega) were added to the normal plasma pool prior to isolation of cfDNA.

Plasma from blood samples from 13 colon cancer patients were obtained from a consecutive series of primary colorectal cancers collected at the Oslo University Hospital between 2010 and 2016. Blood was collected using BD Vacutainer K2E (EDTA) Plus Blood Collection Tubes, and plasma was separated from the cellular fraction by centrifugation at 1600 g for 10 min at 4 °C. The plasma aliquots were stored at − 70 °C. A visual examination of the plasma samples was performed before isolation of cfDNA to ensure no presence of color changes indicating hemolysis.

### Bisulfite conversion

Five different bisulfite conversion kits were compared; EpiJET Bisulfite Conversion Kit (Thermo Scientific), EpiTect Plus DNA Bisulfite Kit (QIAGEN), EZ DNA Methylation-Direct Kit (Zymo Research), Imprint DNA Modification Kit (Sigma-Aldrich) and Premium Bisulfite Kit (Diagenode). An overview of the key characteristics of the bisulfite conversion kits is shown in Table 1. For all kits, the bisulfite conversion was performed according to the manufacturer’s instructions. Carrier RNA was used in the EpiTect kit, whereas bovine serum albumin (BSA) was used as a carrier in the Imprint kit. For the EpiTect kit, the QIAcube Connect (QIAGEN) was used for automated clean-up according to the manufacturer’s instructions.

An input volume of 20 µl was used for all kits, with a range of fragmented RKO DNA; 20, 10, 5, 3, 2, 1 and 0.5 ng. For each input amount, the bisulfite conversion was performed with three technical replicates for all kits. For cfDNA samples from colorectal cancer patients, 25 µl isolated cfDNA was used for bisulfite conversion with the EpiTect kit. An elution volume of 15 µl for the EpiTect kit and 10 µl for the other kits was used, as recommended by the manufacturers. The bisulfite converted DNA was stored at − 20 °C.

### cfDNA isolation

Three different cfDNA isolation kits were compared: Maxwell RSC ccfDNA Plasma Kit (Promega), QIAamp Circulating Nucleic Acid Kit (QIAGEN) and QIAamp MinElute ccfDNA Mini Kit (QIAGEN). An overview of the key characteristics of the cfDNA isolation kits is shown in Table 2. For all kits, the cfDNA isolation was performed according to the manufacturer’s instructions. For the Maxwell kit, the Maxwell RSC Instrument (Promega) was used for automated isolation according to the manufacturer’s instructions. For the CNA and MinElute kits, the QIAcube Connect (QIAGEN) was used for automated clean-up according to the manufacturer’s instructions.

An input volume of 1 ml normal plasma pool was used for all kits, with a range of spiked-in fragmented RKO DNA (10, 5 and 1 ng, as well as 0 ng/only plasma). For each input amount, the cfDNA isolation was performed with three technical replicates for all kits. For plasma samples from colorectal cancer patients, two tubes containing between 2.0 and 3.7 ml plasma in total was used for isolation with the CNA kit, using the 2, 3 or 4 ml protocol. An elution volume of 30 µl for both CNA and MinElute kits and 60 µl for the Maxwell kit was used, within the range recommended by the manufacturers and suitable for downstream analyses. DNA concentration of isolated cfDNA was measured using the Qubit Fluorometer and the Qubit dsDNA High Sensitivity Assay (ThermoFisher Scientific). The isolated cfDNA was stored at − 20 °C.

### Droplet digital PCR

Droplet digital PCR was performed using the QX200 Droplet Digital PCR System (Bio-Rad) as previously described [23]. Two different target assays were used: FAM-labeled target assays for branched chain amino acid transaminase 1 *(BCAT1*) [25] and IKAROS family zinc finger 1 (*IKZF1*) [25]. As a control and for quantification, the VIC-labeled 4Plex assay was used, which comprises of the genes ephrin type A receptor 3 (*EPHA3*), kelch repeat and BTB domain containing 4 (*KBTBD4*), pleckstrin homology and FYVE domain containing 1 (*PLEKHF1*) and synaptotagmin 10 (*SYT10*) [23]. These genes were selected based on their pericentromeric location and stability in regard to copy number variations [23]. The VIC-labeled assay for myogenic differentiation 1 (*MYOD1*) [10] was also used for quantification to compare with the results obtained with 4Plex. Primers were purchased from BioNordika and probes from Life Technologies, and sequences are given in Additional file 2: Table S5. For each experiment, the following control samples were included: two methylation-positive controls (Methylated Human DNA Standard, Zymo Research), one methylation-negative control (Human WGA Non-methylated DNA, Zymo Research) and two non-template controls (NTC; water).

Data analysis was performed using QuantaSoft version 1.7.4.0917 (Bio-Rad). An in-house developed algorithm, PoDCall (https://bioconductor.org/packages/release/bioc/html/PoDCall.html), was applied for positive droplet calling as previously described [23, 33]. Normalized DNA methylation levels were calculated as previously described [34]. Sample exclusion criteria were; (1) low droplet count (total number of droplets < 5000) or (2) low DNA amount (4Plex concentration < 10 copies/µl). In addition, the methylation level was set to zero for samples with only one positive droplet for the target gene. All analyses were performed according to the digital MIQE guidelines [35].

### Fragment length analysis

Fragment length analyses were performed by electrophoretic separation using the 2100 Bioanalyzer Instrument and the 4200 TapeStation Instrument (Agilent). For fragment length analysis of isolated cfDNA, the High Sensitivity DNA Kit (Agilent) with the Bioanalyzer and the Cell-free DNA ScreenTape Assay (Agilent) with the TapeStation were used. The RNA 6000 Pico Kit (Agilent) with the Bioanalyzer was used for assessing the fragment length of bisulfite converted DNA since this is mainly single-stranded. The peak size of the fragment distribution was determined for all assays.

### Supplementary Information

**Additional file 1**: **Fig. S1**. Size distribution of fragmented DNA from RKO cell line. **Fig. S2**. Overview of the workflow for evaluation of bisulfite conversion kits. **Fig. S3**. DNA quantity and DNA recovery after bisulfite conversion using ddPCR with 4Plex and MYOD1 assays. **Fig. S4**. Average peak fragment length of bisulfite converted DNA for bisulfite conversion kits. **Fig. S5**. Overview of the workflow for evaluation of cfDNA isolation kits. **Fig. S6**. Average peak fragment length of isolated cfDNA for cfDNA isolation kits. **Fig. S7**. Fragment length distribution and %cfDNA score for samples for evaluation of contamination of HMW DNA in cfDNA isolation kits. **Fig. S8**. Overview of the workflow for evaluation of combinations of cfDNA isolation and bisulfite conversion kits. **Fig. S9**. Average peak fragment length of bisulfite converted cfDNA for combinations of cfDNA isolation and bisulfite conversion kits. **Fig. S10**. Fragment length distribution and %cfDNA score of isolated cfDNA from colorectal cancer plasma samples.

**Additional file 2**: **Table S1**. Ranking and scoring of bisulfite conversion kits using the 4Plex ddPCR control. **Table S2**. Ranking and scoring of bisulfite conversion kits using the MYOD1 ddPCR control. **Table S3**. Ranking and scoring of combinations of cfDNA isolation and bisulfite conversion kits . **Table S4**. Comparison of the amount of isolated cfDNA, the %cfDNA score and the detection of DNA methylation of *BCAT1* and/or *IKZF1*. **Table S5**. Sequences of primers and probes used for ddPCR.

## Data Availability

All data that support the findings of this study are either available in the article and in the supplementary materials, or from the corresponding author upon reasonable request.

## Abbreviations

cfDNA: Cell-free deoxyribonucleic acid
ddPCR: Droplet digital polymerase chain reaction
DNA: Deoxyribonucleic acid
HMW: High molecular weight
