# Supplementary material for: Evaluation of commercial kits for isolation and bisulfite conversion of circulating cell-free tumor DNA from blood
Source: Clin Epigenetics. 2023 Sep 14;15:151. doi: 10.1186/s13148-023-01563-0 (PMC10503171; doi:10.1186/s13148-023-01563-0)
Supplement: Supplementary file 1 — Additional file 1: Fig. S1. Size distribution of fragmented DNA from RKO cell line. Fig. S2. Overview of the workflow for evaluation of bisulfite conversion kits. Fig. S3. DNA quantity and DNA recovery after bisulfite conversion using ddPCR with 4Plex and MYOD1 assays. Fig. S4. Average peak fragment length of bisulfite converted DNA for bisulfite conversion kits. Fig. S5. Overview of the workflow for evaluation of cfDNA isolation kits. Fig. S6. Average peak fragment length of isolated cfDNA for cfDNA isolation kits. Fig. S7. Fragment length distribution and %cfDNA score for samples for evaluation of contamination of HMW DNA in cfDNA isolation kits. Fig. S8. Overview of the workflow for evaluation of combinations of cfDNA isolation and bisulfite conversion kits. Fig. S9. Average peak fragment length of bisulfite converted cfDNA for combinations of cfDNA isolation and bisulfite conversion kits. Fig. S10. Fragment length distribution and %cfDNA score of isolated cfDNA from colorectal cancer plasma samples. [file 13148_2023_1563_MOESM1_ESM.pptx]

## Slide 1
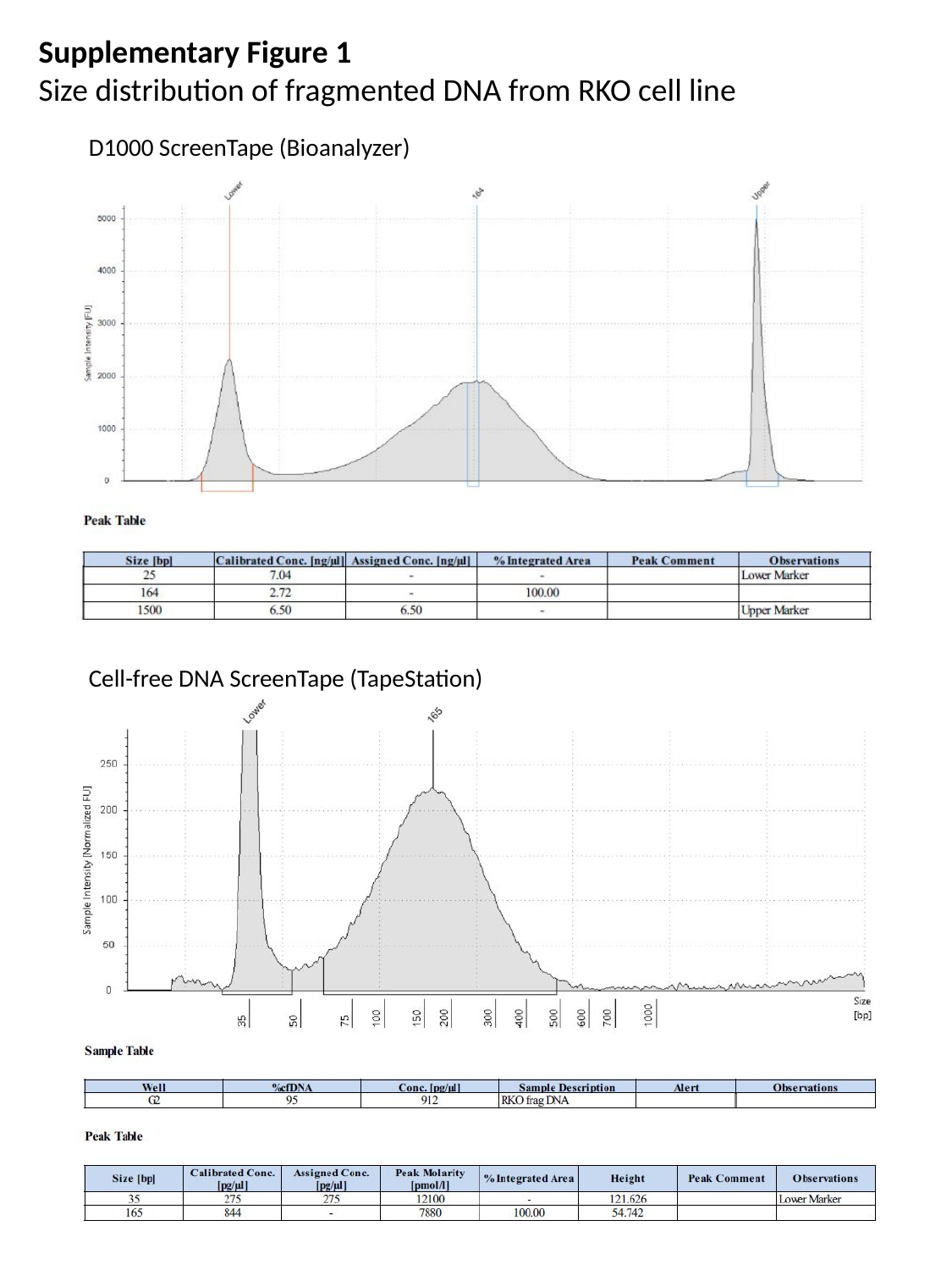

Supplementary Figure 1
Size distribution of fragmented DNA from RKO cell line
D1000 ScreenTape (Bioanalyzer)
Cell-free DNA ScreenTape (TapeStation)

## Slide 2
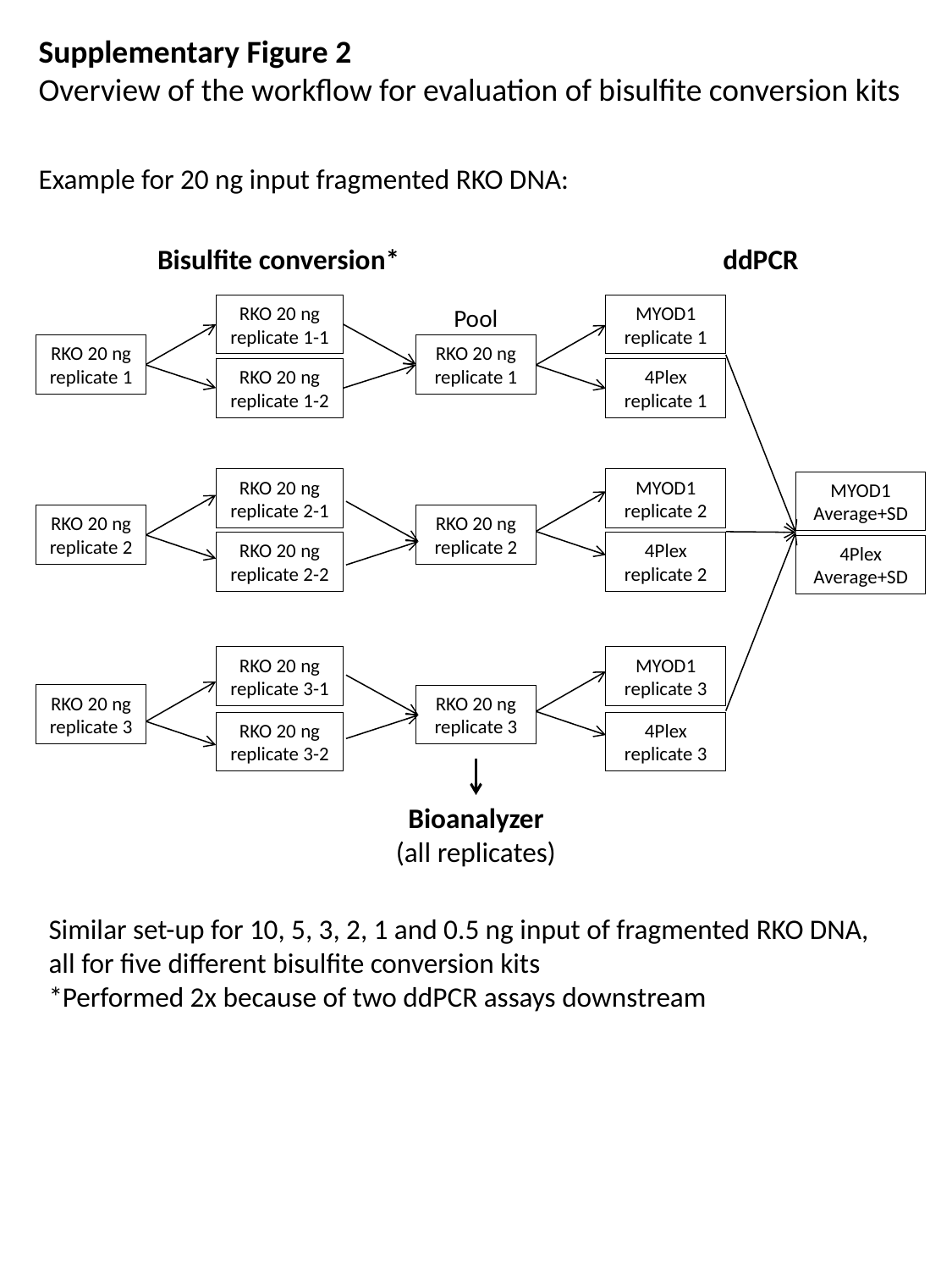

Supplementary Figure 2
Overview of the workflow for evaluation of bisulfite conversion kits
Example for 20 ng input fragmented RKO DNA:
Bisulfite conversion*
ddPCR
RKO 20 ng replicate 1-1
MYOD1
replicate 1
Pool
RKO 20 ng replicate 1
RKO 20 ng replicate 1
RKO 20 ng replicate 1-2
4Plex
replicate 1
RKO 20 ng replicate 2-1
MYOD1 replicate 2
MYOD1
Average+SD
RKO 20 ng replicate 2
RKO 20 ng replicate 2
RKO 20 ng replicate 2-2
4Plex replicate 2
4Plex
Average+SD
RKO 20 ng replicate 3-1
MYOD1 replicate 3
RKO 20 ng replicate 3
RKO 20 ng replicate 3
RKO 20 ng replicate 3-2
4Plex replicate 3
Bioanalyzer
(all replicates)
Similar set-up for 10, 5, 3, 2, 1 and 0.5 ng input of fragmented RKO DNA, all for five different bisulfite conversion kits
*Performed 2x because of two ddPCR assays downstream

## Slide 3
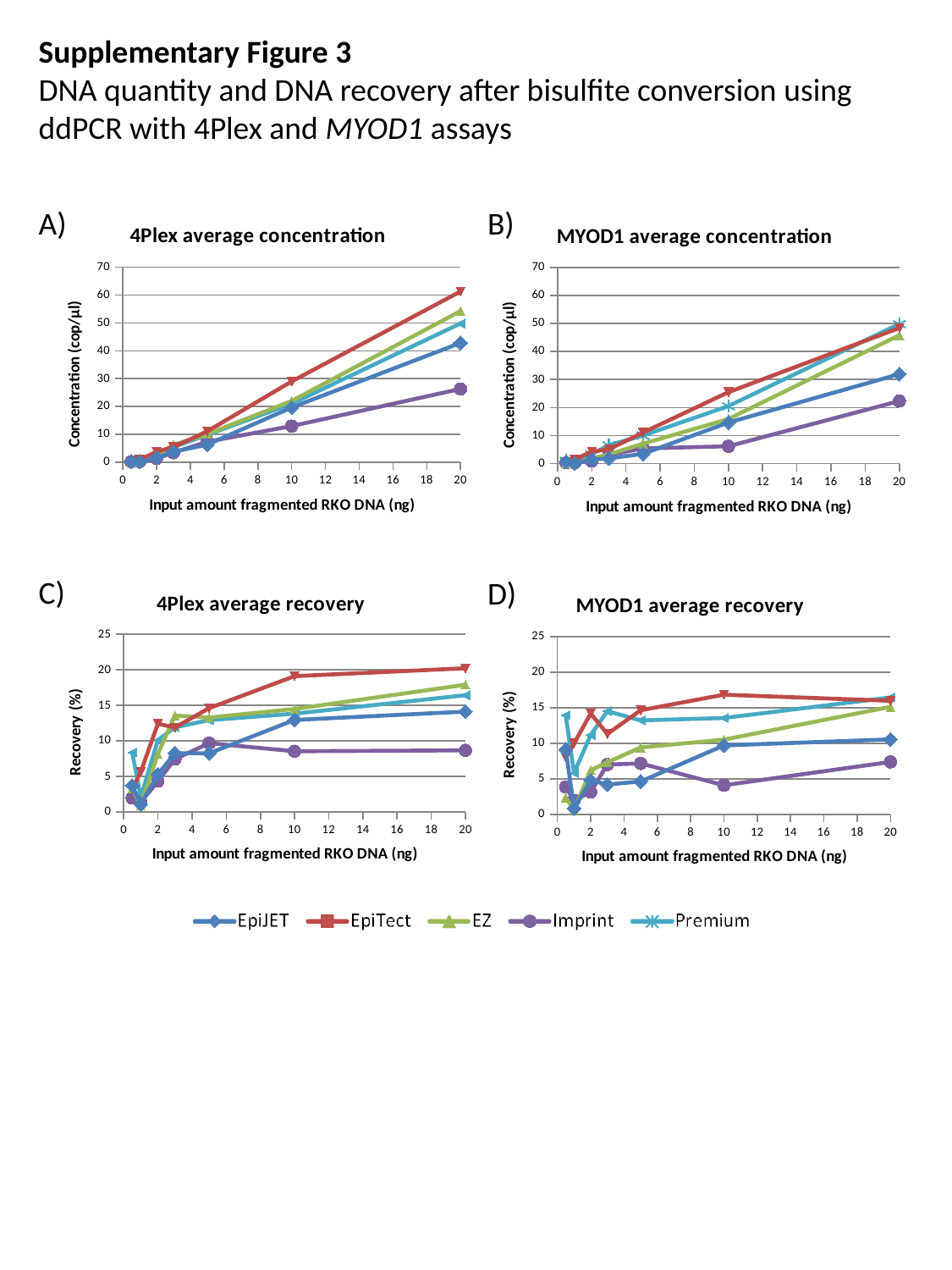

Supplementary Figure 3
DNA quantity and DNA recovery after bisulfite conversion using ddPCR with 4Plex and MYOD1 assays
A)
B)
### Chart: 4Plex average concentration
| Category | EpiJET | EpiTect | EZ | Imprint | Premium |
|---|---|---|---|---|---|
### Chart: MYOD1 average concentration
| Category | EpiJET | EpiTect | EZ | Imprint | Premium |
|---|---|---|---|---|---|C)
D)
### Chart: 4Plex average recovery
| Category | EpiJET | EpiTect | EZ | Imprint | Premium |
|---|---|---|---|---|---|
### Chart: MYOD1 average recovery
| Category | EpiJET | EpiTect | EZ | Imprint | Premium |
|---|---|---|---|---|---|

## Slide 4
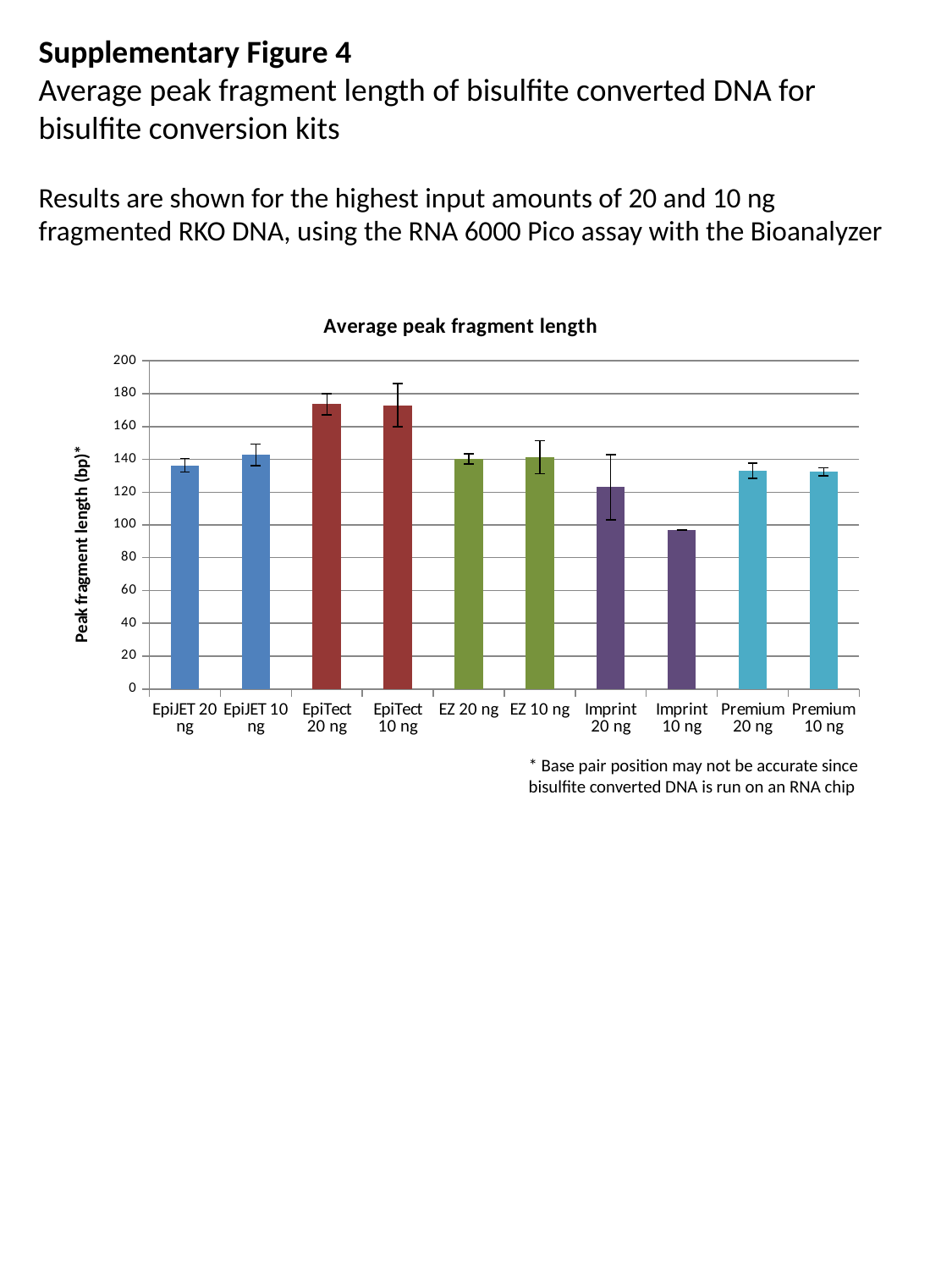

Supplementary Figure 4
Average peak fragment length of bisulfite converted DNA for bisulfite conversion kits
Results are shown for the highest input amounts of 20 and 10 ng fragmented RKO DNA, using the RNA 6000 Pico assay with the Bioanalyzer
### Chart: Average peak fragment length
| Category | Peak size |
|---|---|
| EpiJET 20 ng | 136.33333333333337 |
| EpiJET 10 ng | 142.66666666666663 |
| EpiTect 20 ng | 173.66666666666663 |
| EpiTect 10 ng | 173.0 |
| EZ 20 ng | 140.33333333333337 |
| EZ 10 ng | 141.33333333333337 |
| Imprint 20 ng | 123.0 |
| Imprint 10 ng | 97.0 |
| Premium 20 ng | 133.0 |
| Premium 10 ng | 132.33333333333337 |* Base pair position may not be accurate since bisulfite converted DNA is run on an RNA chip

## Slide 5
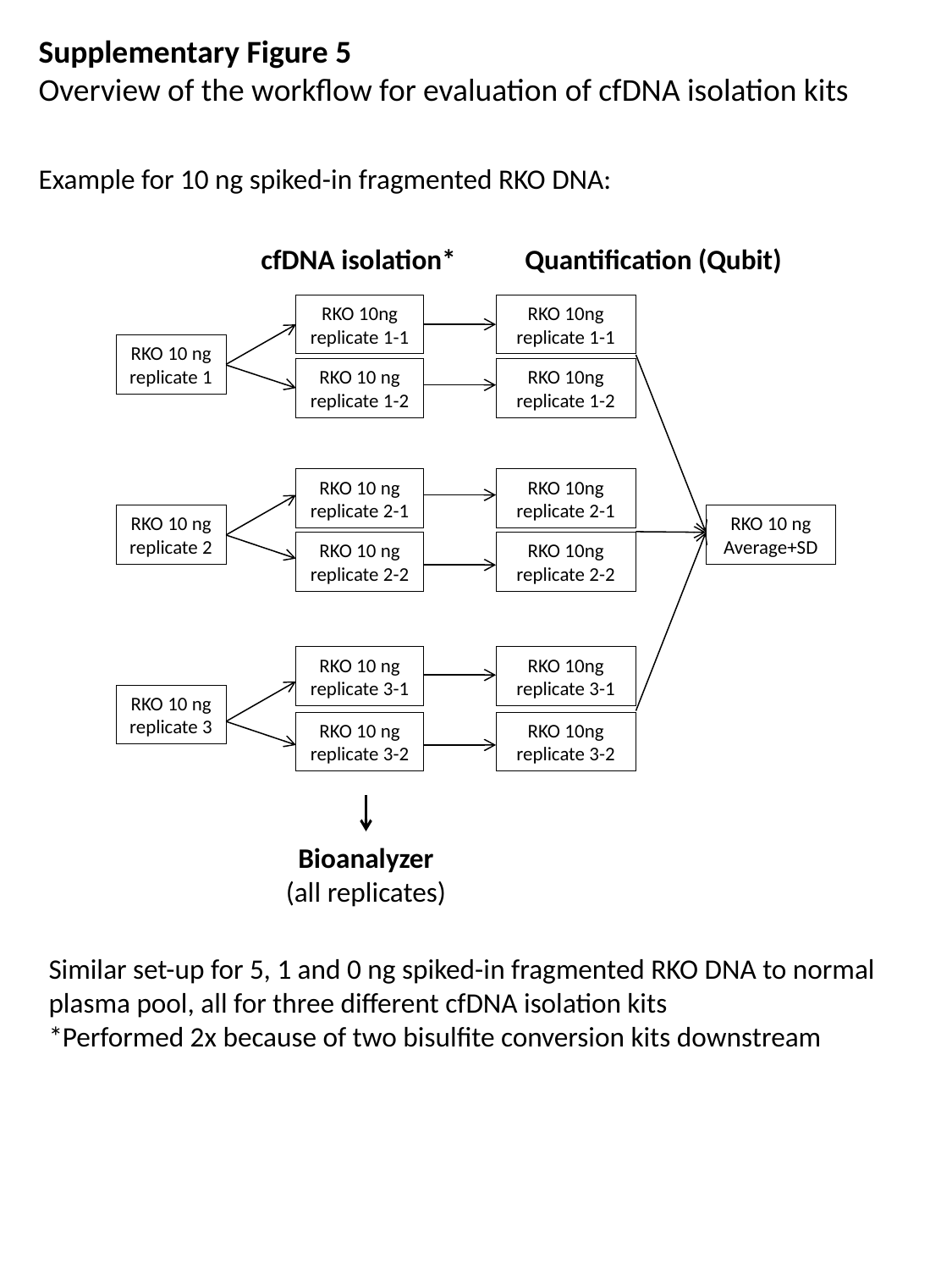

Supplementary Figure 5
Overview of the workflow for evaluation of cfDNA isolation kits
Example for 10 ng spiked-in fragmented RKO DNA:
cfDNA isolation*
Quantification (Qubit)
RKO 10ng replicate 1-1
RKO 10ng replicate 1-1
RKO 10 ng replicate 1
RKO 10 ng replicate 1-2
RKO 10ng replicate 1-2
RKO 10 ng replicate 2-1
RKO 10ng replicate 2-1
RKO 10 ng replicate 2
RKO 10 ng
Average+SD
RKO 10 ng replicate 2-2
RKO 10ng replicate 2-2
RKO 10 ng replicate 3-1
RKO 10ng replicate 3-1
RKO 10 ng replicate 3
RKO 10 ng replicate 3-2
RKO 10ng replicate 3-2
Bioanalyzer
(all replicates)
Similar set-up for 5, 1 and 0 ng spiked-in fragmented RKO DNA to normal plasma pool, all for three different cfDNA isolation kits
*Performed 2x because of two bisulfite conversion kits downstream

## Slide 6
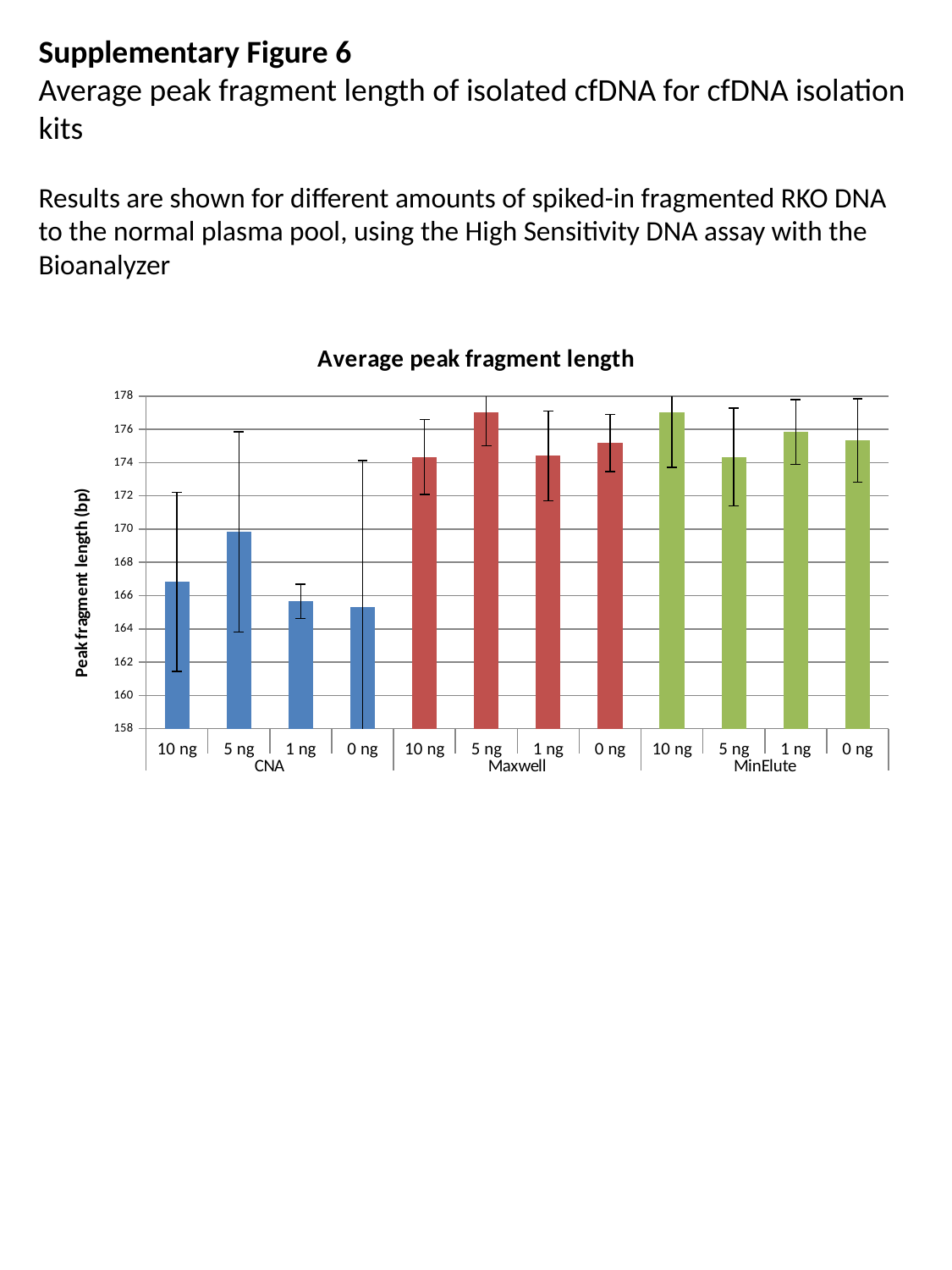

Supplementary Figure 6
Average peak fragment length of isolated cfDNA for cfDNA isolation kits
Results are shown for different amounts of spiked-in fragmented RKO DNA to the normal plasma pool, using the High Sensitivity DNA assay with the Bioanalyzer
### Chart: Average peak fragment length
| Category | Mean peak |
|---|---|
| 10 ng | 166.83333333333334 |
| 5 ng | 169.83333333333334 |
| 1 ng | 165.66666666666666 |
| 0 ng | 165.33333333333334 |
| 10 ng | 174.33333333333334 |
| 5 ng | 177.0 |
| 1 ng | 174.4 |
| 0 ng | 175.16666666666666 |
| 10 ng | 177.0 |
| 5 ng | 174.33333333333334 |
| 1 ng | 175.83333333333334 |
| 0 ng | 175.33333333333334 |

## Slide 7
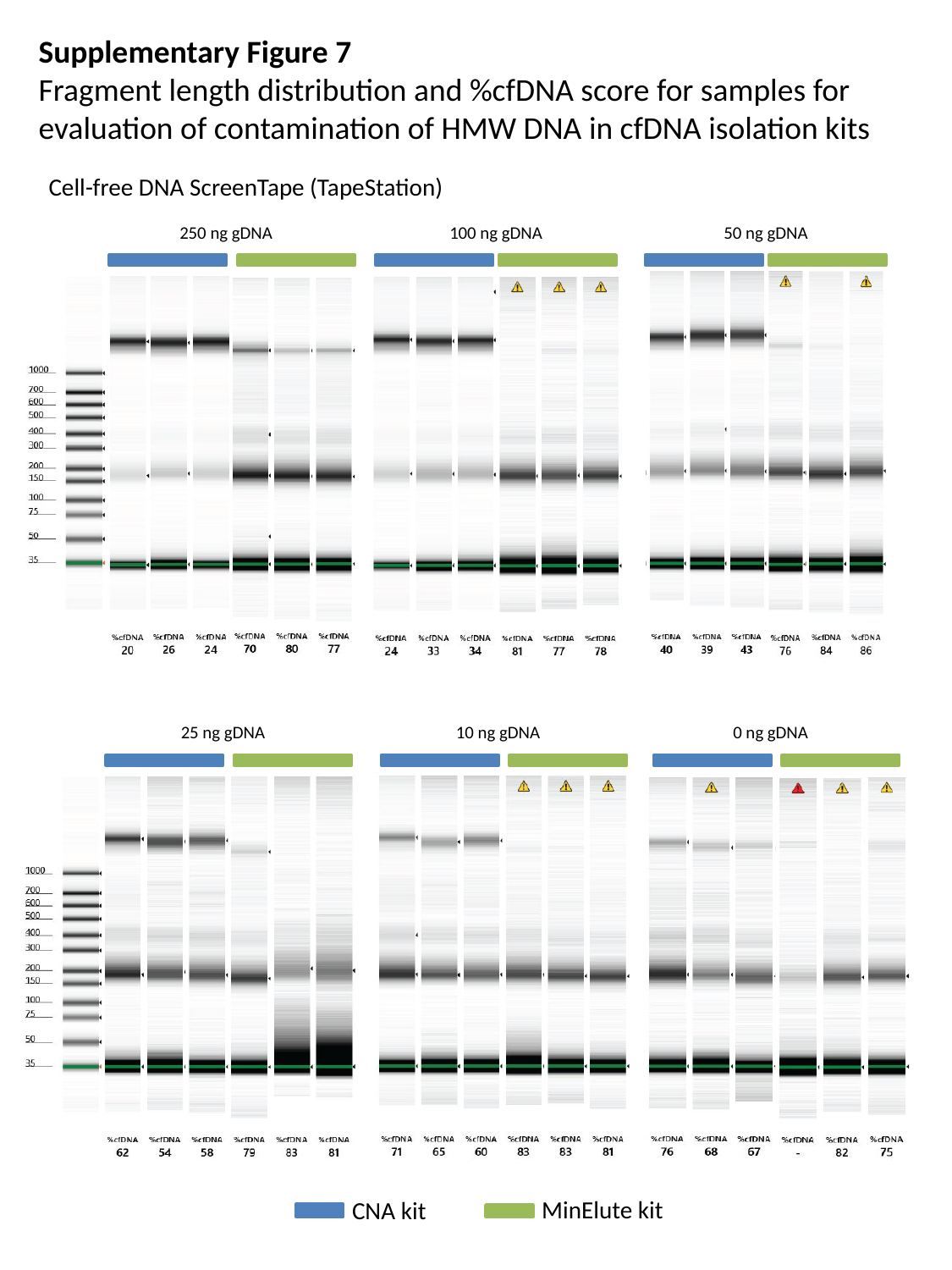

Supplementary Figure 7
Fragment length distribution and %cfDNA score for samples for evaluation of contamination of HMW DNA in cfDNA isolation kits
Cell-free DNA ScreenTape (TapeStation)
250 ng gDNA
100 ng gDNA
50 ng gDNA
25 ng gDNA
10 ng gDNA
0 ng gDNA
MinElute kit
CNA kit

## Slide 8
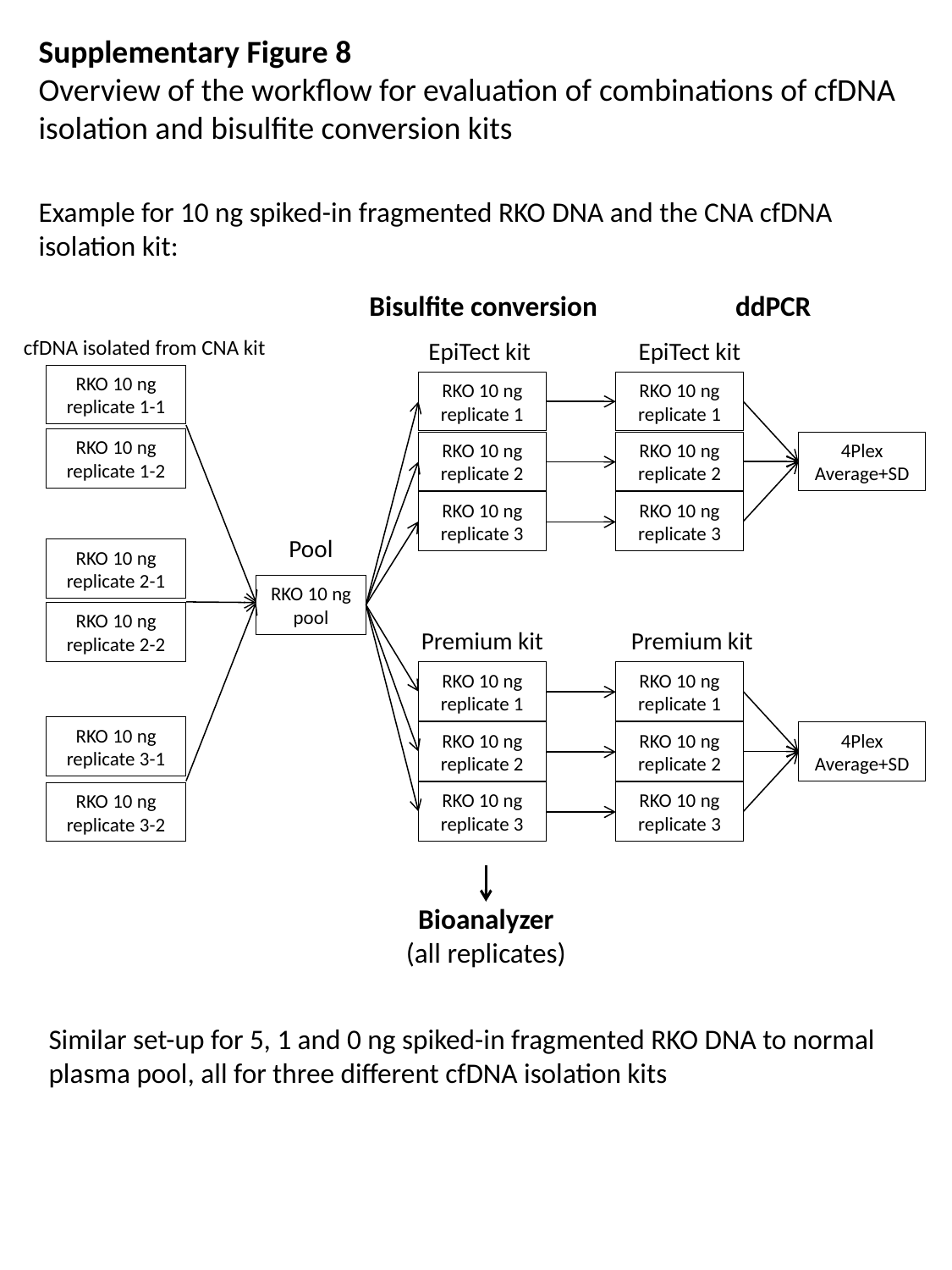

Supplementary Figure 8
Overview of the workflow for evaluation of combinations of cfDNA isolation and bisulfite conversion kits
Example for 10 ng spiked-in fragmented RKO DNA and the CNA cfDNA isolation kit:
Bisulfite conversion
ddPCR
cfDNA isolated from CNA kit
EpiTect kit
EpiTect kit
RKO 10 ng replicate 1-1
RKO 10 ng replicate 1
RKO 10 ng replicate 1
RKO 10 ng replicate 1-2
RKO 10 ng replicate 2
RKO 10 ng replicate 2
4Plex
Average+SD
RKO 10 ng replicate 3
RKO 10 ng replicate 3
Pool
RKO 10 ng replicate 2-1
RKO 10 ng
pool
RKO 10 ng replicate 2-2
Premium kit
Premium kit
RKO 10 ng replicate 1
RKO 10 ng replicate 1
RKO 10 ng replicate 3-1
RKO 10 ng replicate 2
RKO 10 ng replicate 2
4Plex
Average+SD
RKO 10 ng replicate 3
RKO 10 ng replicate 3
RKO 10 ng replicate 3-2
Bioanalyzer
(all replicates)
Similar set-up for 5, 1 and 0 ng spiked-in fragmented RKO DNA to normal plasma pool, all for three different cfDNA isolation kits

## Slide 9
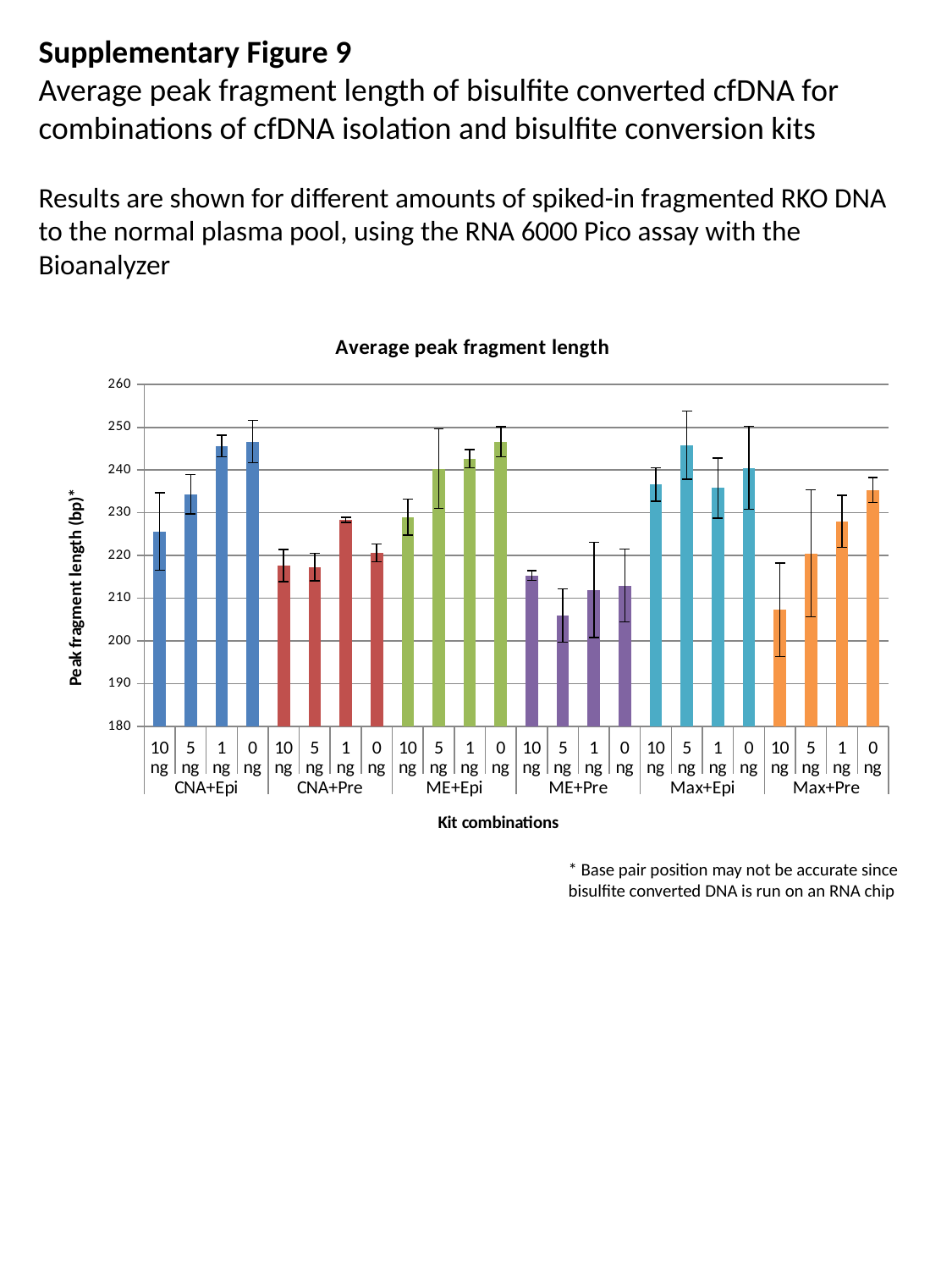

Supplementary Figure 9
Average peak fragment length of bisulfite converted cfDNA for combinations of cfDNA isolation and bisulfite conversion kits
Results are shown for different amounts of spiked-in fragmented RKO DNA to the normal plasma pool, using the RNA 6000 Pico assay with the Bioanalyzer
### Chart: Average peak fragment length
| Category | Mean peak |
|---|---|
| 10 ng | 225.66666666666666 |
| 5 ng | 234.33333333333334 |
| 1 ng | 245.66666666666666 |
| 0 ng | 246.66666666666666 |
| 10 ng | 217.66666666666666 |
| 5 ng | 217.33333333333334 |
| 1 ng | 228.33333333333334 |
| 0 ng | 220.66666666666666 |
| 10 ng | 229.0 |
| 5 ng | 240.33333333333334 |
| 1 ng | 242.66666666666666 |
| 0 ng | 246.66666666666666 |
| 10 ng | 215.33333333333334 |
| 5 ng | 206.0 |
| 1 ng | 212.0 |
| 0 ng | 213.0 |
| 10 ng | 236.6 |
| 5 ng | 245.83333333333334 |
| 1 ng | 235.83333333333334 |
| 0 ng | 240.5 |
| 10 ng | 207.33333333333334 |
| 5 ng | 220.5 |
| 1 ng | 228.0 |
| 0 ng | 235.33333333333334 |* Base pair position may not be accurate since bisulfite converted DNA is run on an RNA chip

## Slide 10
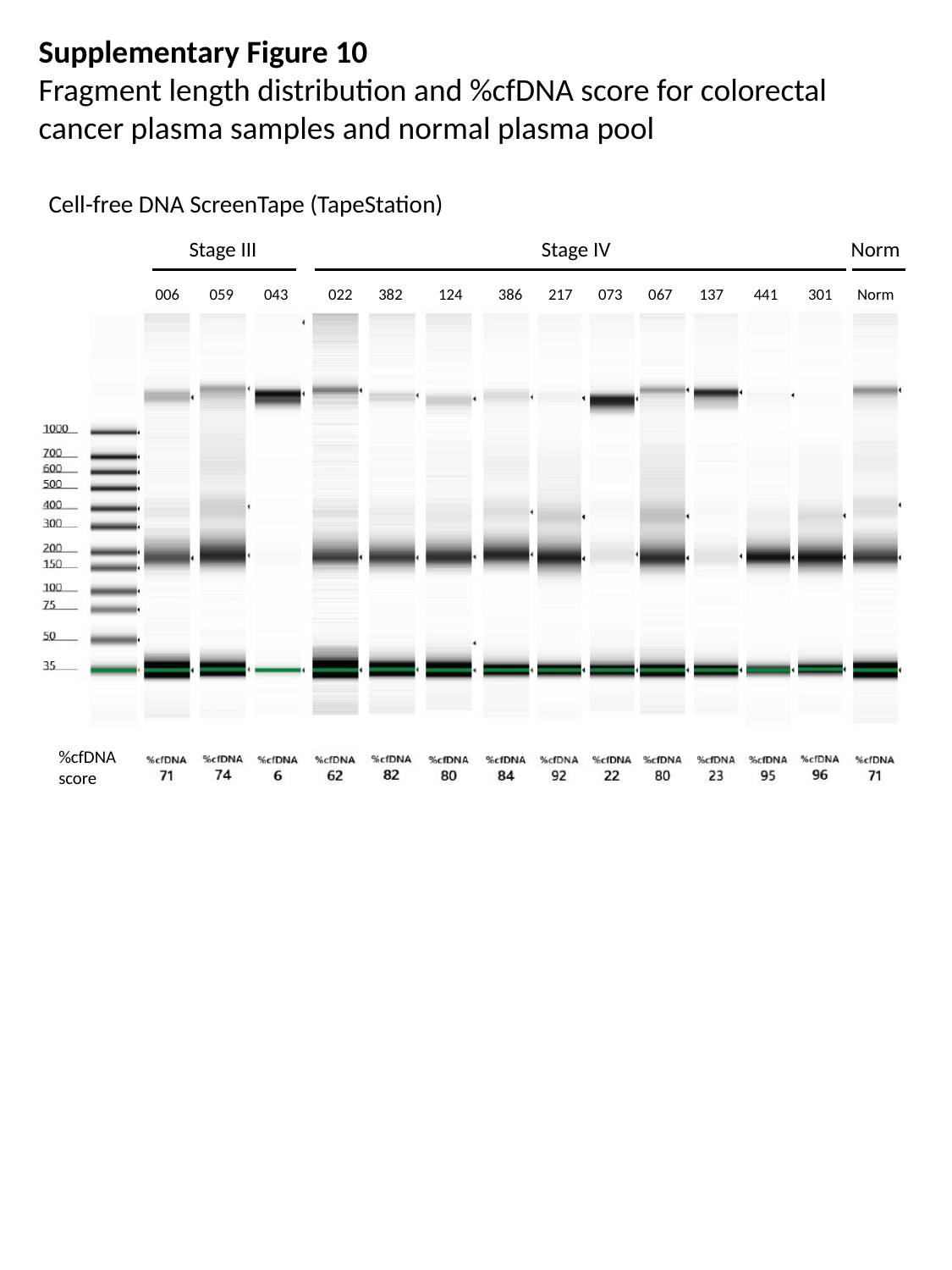

Supplementary Figure 10
Fragment length distribution and %cfDNA score for colorectal cancer plasma samples and normal plasma pool
Cell-free DNA ScreenTape (TapeStation)
Stage III
Stage IV
Norm
006
059
043
022
382
124
386
217
073
067
137
441
301
Norm
%cfDNA score
